# Examining Mental Workload in a Spatial Navigation Transfer Game via Functional near Infrared Spectroscopy

**DOI:** 10.3390/brainsci11010045

**Published:** 2021-01-04

**Authors:** Tamara Galoyan, Kristen Betts, Hovag Abramian, Pratusha Reddy, Kurtulus Izzetoglu, Patricia A. Shewokis

**Affiliations:** 1 Department of Educational Psychology, College of Education, The University of Utah, Salt Lake City, UT 84112, USA; 2 School of Education, Drexel University, Philadelphia, PA 19104, USA; ksb23@drexel.edu; 3 College of Science and Engineering, American University of Armenia, Yerevan 0019, Armenia; hovag.abramian@aua.am; 4 School of Biomedical Engineering, Science and Health Systems, Drexel University, Philadelphia, PA 19104, USA; ylr26@drexel.edu (P.R.); ki25@drexel.edu (K.I.); pas38@drexel.edu (P.A.S.)

**Keywords:** mental workload, cognitive load, problem solving, spatial navigation game, functional near-infrared spectroscopy (fNIRS)

## Abstract

The goal of this study was to examine the effects of task-related variables, such as the difficulty level, problem scenario, and experiment week, on performance and mental workload of 27 healthy adult subjects during problem solving within the spatial navigation transfer (SNT) game. The study reports task performance measures such as total time spent on a task (TT) and reaction time (RT); neurophysiological measures involving the use of functional near-infrared spectroscopy (fNIRS); and a subjective rating scale for self-assessment of mental workload (NASA TLX) to test the related hypothesis. Several within-subject repeated-measures factorial ANOVA models were developed to test the main hypothesis. The results revealed a number of interaction effects for the dependent measures of TT, RT, fNIRS, and NASA TLX. The results showed (1) a decrease in TT and RT across the three levels of difficulty from Week 1 to Week 2; (2) an increase in TT and RT for high and medium cognitive load tasks as compared to low cognitive load tasks in both Week 1 and Week 2; (3) an overall increase in oxygenation from Week 1 to Week 2. These findings confirmed that both the behavioral performance and mental workload were sensitive to task manipulations.

## 1. Introduction

In the human brain, the prefrontal cortex (PFC) plays an important role in executive functions involving cognitive processes such as problem solving, decision making, planning, and working memory [1,2,3]. To understand the complex nature of these cognitive processes within the PFC area, it is important to examine mental workload that is taxed by various cognitive tasks. Mental workload was defined as the cognitive and psychological effort that is required from a subject to complete a given task [4,5]. Previous studies indicated that mental workload is sensitive to task-related features including the difficulty level of the task, the order in which the tasks were presented (e.g., blocked vs. random practice), and the type of the task (e.g., learning vs. transfer task) [6,7,8].

Some researchers described the concept of mental workload as a section within broader cognitive load theory (CLT) [9]. CLT, developed in the 1980s, informs instructional design by proposing instructional strategies that are based on human cognitive architecture and can be used to enhance cognitive processes [10,11,12]. CLT aims at maximizing working memory resources to enhance learning and handling complex learning tasks which can exert a heavy cognitive load on the memory system [13,14,15,16]. According to CLT, working memory load can be influenced by three types of cognitive load, namely intrinsic cognitive load, extraneous cognitive load, and germane cognitive load [13,14,15]. Intrinsic cognitive load is characterized by the intrinsic nature of the task itself and depends on the number of elements to be processed simultaneously in working memory. This number, in its turn, depends on the level of element interactivity of the learning task, or, in other words, the extent to which the constituent elements of a task can or cannot be learned in isolation. Extraneous cognitive load is associated with the instructional strategies used to present the task to the learner (e.g., visual, audio) and is not directly related to the intrinsic nature of the task. Germane load is directly associated with learning and is described in terms of the cognitive resources used to learn something.

While subjective rating scales were widely used as a common technique for assessing cognitive load [17,18], Sweller and colleagues [10] called for the need of further research into physiological techniques (e.g., functional magnetic resonance imaging (fMRI)) of measuring cognitive load. Specifically, functional near-infrared spectroscopy (fNIRS) [19,20] is a promising noninvasive and portable technology that allows for monitoring PFC activation during complex cognitive tasks in natural settings [21,22,23,24]. Previous studies demonstrated that fNIRS can indicate various levels of mental workload changes associated with varying difficulty levels of the tasks [22,25,26].

In this study, we used the fNIRS to assess mental workload in the context of general problem solving in the spatial navigation transfer (SNT) game. The SNT game was designed with a focus on complex cognitive and visuospatial tasks involving analogical reasoning, spatial navigation, planning, and decision making, all of which have been associated with executive functions where the PFC region plays a role [1,2,3,27]. The game allowed for the manipulation of intrinsic cognitive load by varying the difficulty level of the analogous tasks as well as the manipulation of extraneous cognitive load by varying the contextual (surface) features of the task such as the problem scenarios. Previous studies provide evidence of successful fNIRS deployment for monitoring hemodynamic changes within complex cognitive and visuospatial tasks such as air traffic control [26] or maze navigation [28].

The goal of this study was to examine the effects of task-related variables on performance and mental workload during problem solving within the SNT game, which is a serious gaming approach for a task design to study cognitive load. The task-related variables under examination included the level of difficulty of the task (Level) with three levels (low cognitive load, medium cognitive load, and high cognitive load), the type of the problem scenario (Problem) with two levels (School Bus and Ambulance), and the week in which the task was presented (Week) with two levels (Week 1 and Week 2). The study investigated task performance measures such as total time spent on a task (TT) and reaction time (RT); neurophysiological measures involving the use of the fNIRS and a subjective rating scale for self-assessment of mental workload (NASA TLX) [17]. The overarching hypothesis was that there are main effects and interaction (three-way and/or two-way) effects between Week, Problem, and Level on mental workload as measured by Oxygenation (OXY (µV)) and perceived mental workload as measured by NASA TLX. Prior to testing this main hypothesis, behavioral performances were also analyzed in terms of reaction time and total time over Week, Problem and Level for the main effect and interactions among them.

## 2. Materials and Methods

### 2.1. Spatial Navigation Transfer (SNT) Game Tasks

The SNT game was designed and developed by the researchers of this study using CLT as an analytical framework. Specifically, the game allowed for manipulation of two types of cognitive load: intrinsic cognitive load was manipulated by varying the difficulty levels of the tasks and the extraneous load was manipulated by varying the contextual features of the game such as the problem scenarios. 

**Figure 1:**
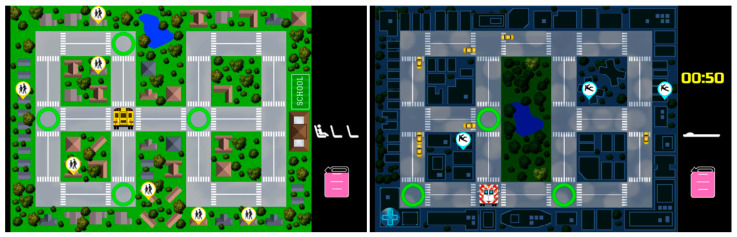
Screenshots of two high cognitive load tasks from the School Bus (left) and Ambulance (right) scenarios.

The game contained two well-structured problems represented by two analogous scenarios: the School Bus problem and the Ambulance problem (See Figure 1), which were similar at a deep, structural level (e.g., navigating the map to reach certain destinations while accounting for the constraints) but differed at a surface, contextual level (e.g., driving a school bus at daytime vs. driving an ambulance at night). Each of the two problem scenarios included nine analogical tasks of three levels of difficulty: low, medium, and high cognitive load. The difficulty level of each task for both problem scenarios was defined by the number of constraints such as a limited amount of fuel, limited amount of time, limited number of seats, and increasing traffic. In both problem scenarios, the subjects were required to perform a number of cognitive and visuospatial tasks. For instance, in the School Bus scenario, participants assumed the role of a school bus driver whose task was to navigate the game map to collect students from different locations and drive them to school while accounting for the limited seating and fuel. In the Ambulance scenario, participants assumed the role of an ambulance driver whose task was to collect patients from different locations on the game map and take them to the hospital while accounting for limited time and fuel. The tasks within each level were preceded by detailed instruction pages containing the description of the tasks and the type of constraints contained in the tasks. The game had been pilot tested with six subjects representing the target population. Those who participated in the pilot were not included in this study. The pilot testing allowed for the validation of the difficulty levels of the tasks, as well as modifications of certain game design features (e.g., clarifying the in-game instructions and feedback, editing the visuals, etc.). 

### 2.2. Functional Near Infrared Spectroscopy (fNIRS)

**Figure 2:**
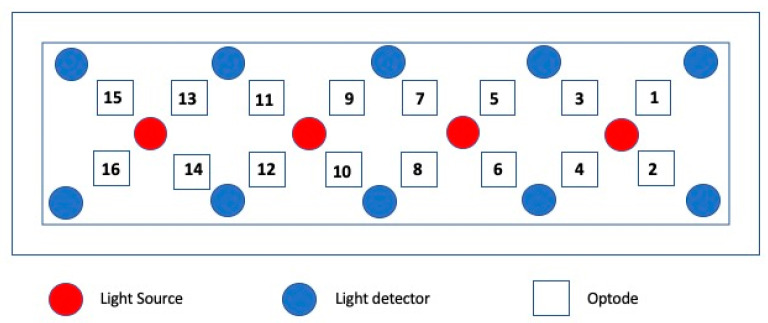
Functional near-infrared spectroscopy (fNIRS) sensor layout with 16 optodes.

Participants’ brain activity changes during the SNT gameplay were assessed using the functional near infrared spectroscopy (fNIRS). As a field-deployable non-invasive optical brain monitoring technology, fNIRS provides a measure of cerebral hemodynamics in response to sensory, motor, or cognitive activation. It offers greater flexibility for deployment in dynamic environments relative to other neuroimaging modalities since it allows for continuous and localized cortical activity monitoring for physiological and psychological assessment while requiring a small and portable footprint, low initial investment, and near-zero runtime costs [19,20,22,23]. A commercially available research-grade fNIRS system (fNIR Devices LLC, Potomac, MD) was utilized to conduct neurophysiological assessment tests. This research grade, 16-optode system allows to scan whole forehead and provides measures for oxygenated (HbO) and deoxygenated hemoglobin (HbR) concentration changes from the prefrontal cortex region, the area associated with higher cognitive functions, attention, working memory, decision making, and problem solving. The fNIRS sensor has 4 light emitting diodes (LEDs) and 10 photodetectors resulting in an array of 16 channels with 2 Hz. sampling rate (see Figure 2).

### 2.3. Participants and Experiment Protocol

A total of 27 right-handed and healthy adults (13 males and 14 females) ranging from 18 to 40 years old (26–30 being the prevalent age group) volunteered for participation in this study. The volunteers were graduate students majoring in education, neuroscience, psychology, or business at a four-year private university located in the northeast of the US (see Appendix A for the selection criteria). IRB Protocol Number: 1806006395.

**Figure 3:**
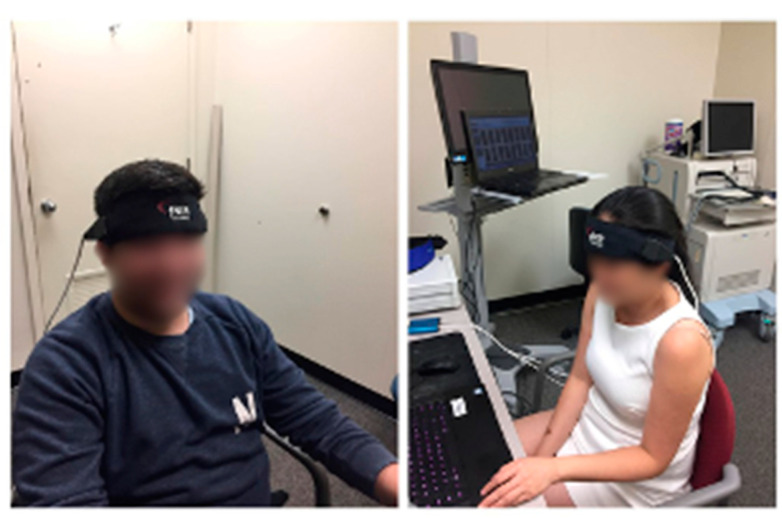
Participants wearing the fNIRS sensor pad.

Prior to the experiment, all participants signed informed written consent forms approved by the Drexel University Human Subjects Institution Review Board. This was followed by the completion of a demographic questionnaire administered using the Qualtrics online survey tool. The questionnaire collected information about the participants’ age, gender, and disciplinary area (reported earlier in this section). One week after the completion of the survey, the participants were invited to the experiment lab located at the target university to play the SNT game during two sessions across two weeks. At the beginning of session one, the participants received verbal introduction familiarizing them with the experiment protocol and the SNT game. In both sessions, the participants were asked to follow the in-game instructions and try to keep calm and minimize their movements during the experiment. Next, the fNIRS sensor pad was placed on the participant’s forehead (see Figure 3). Afterwards, the participants were instructed to login to the game by entering their unique user ID. The user IDs helped to match participant survey responses and the experimental data collected in two sessions across two weeks. The game was played online on a 14-inch laptop. The participants were asked to use a mouse to navigate the game.

**Figure 4:**
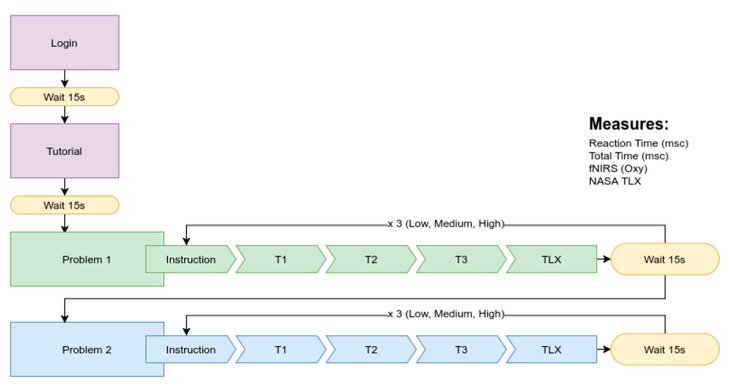
Spatial navigation transfer (SNT) game tasks and measures.

**Figure 5:**
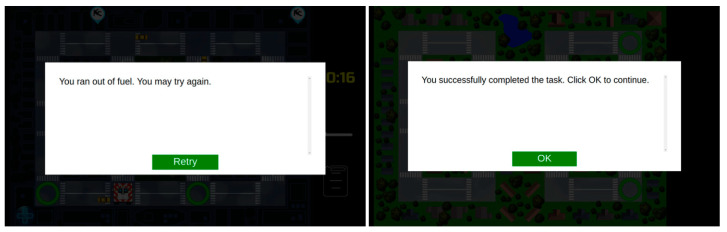
Screenshots of two pages prompting participants to repeat the task (left) and to continue (right).

To determine each participant’s cognitive baseline (fNIRS measures), a 15-s resting period was recorded while the participants were relaxed and still. The game started with a tutorial providing them with step-by-step instructions on how to navigate the game and collect baseline information on the participants’ behavioral performance in the context of the game (see Appendix B). The tutorial presented the Farm problem, analogous to the Ambulance and School Bus problems, where the task was to navigate the map to collect milk from different locations and bring it back to the farm (see Appendix C). In each week, the participants completed 18 tasks which were presented in randomized blocks of three tasks (T1, T2, T3) (see Figure 4). The three tasks within a block were of the same level of difficulty. After completing each block, the participants were presented with the NASA TLX screen asking them to rate the perceived mental workload of the tasks. If the participant failed a task, they were prompted to repeat the same task a second time. If they failed a second time, they could not repeat the task and were prompted to proceed to the next task (see Figure 5). In Week 2, the tasks were introduced in a different order compared to Week 1. Another difference was that in Week 2, some task features (e.g., the number of collectables, the amount of fuel, the location of the collectables, etc.) within each level were manipulated to make them slightly more challenging as compared to Week 1. For example, for the School Bus scenario, in Week 2, the participants had an increased number of collectables. For the Ambulance scenario, in Week 2, the participants had a decreased amount of in-game time available. This slight manipulation ensured that transfer of learning could potentially occur between highly similar rather than the same tasks since contextual similarity is a necessary precondition for transfer [29,30,31]. 

### 2.4. Data Analysis

#### 2.4.1. fNIRS Data Processing

The first step for the fNIRS signals was to apply the noise removal procedures to tease out artifacts due to movement and systemic changes. Different sources of noise might include head movements, physiological signals such as respiration and heart rate, as well as instrument-related and environmental noise [20,27]. After inspecting the data, the saturated channels were rejected, then raw light intensity was filtered by a low-pass finite impulse response (FIR) filter with a cut-off frequency of 0.1 Hz to eliminate the effects of any physiological and/or non-physiological noise sources. Next, relative changes of concentrations in oxygenated hemoglobin (oxy-Hb) and deoxygenated hemoglobin (deoxy-Hb) were calculated with modified Beer−Lambert law, using the baseline recorded at the beginning of the experimental data collection [1]. Blood oxygenation (OXY) was calculated using HbO−HbR and used in subsequent statistical analyses. Mean (average) statistics for OXY were calculated across the right channels (right dorsolateral (RDLPFC); right anterior (RANTPFC)), left channels (left dorsolateral (LDLPFC); left anterior (LANTPFC)). Previous studies that used average OXY as a dependent measure in complex cognitive tasks demonstrated its sensitivity to task difficulty levels and established validity of average OXY for measuring cortical changes associated with cognitive workload, e.g., [32,33]. Additionally, in an fNIRS study conducted by Liang and colleagues [1], medium effect sizes for OXY and oxy-Hb [d = 0.44–0.76] and small effect sizes [d = 0.26–0.28] for deoxy-Hb and total hemoglobin (HbT) were reported, suggesting that OXY and oxy-Hb were more sensitive to workload changes compared to the other two biomarkers. In addition, different brain regions within PFC reported varying sensitivity levels to workload changes associated with complex cognitive tasks [1,25,28,32]. For instance, the fNIRS study by Izzetoglu and colleagues [32] showed that the left anterior medial PFC was more sensitive to workload changes in cognitive tasks involving a virtual flight simulator. It is also known that the left DLPFC supports cognitive processes of reasoning, problem solving, and recognizing specific features while the right DLPFC supports the cognitive processes of planning and decision making [34,35,36]. Given that the SNT game tasks involved cognitive processes that require functions of PFC from both hemispheres, our statistical analyses involved measures from both the left (LDLPFC, LANTPFC) and the right (RDLPFC, RANTPFC) regions.

#### 2.4.2. Statistical Analyses

The independent variables included experiment week (Week) with two levels (Week 1 and Week 2), problem scenario (Problem) with two levels (School Bus and Ambulance), and difficulty level (Level) with three levels (low cognitive load, medium cognitive load, and high cognitive load). The dependent variables included performance operationalized in terms of reaction time (RT) and total time (TT), and mean changes in oxygenation (OXY) for four regions of interest within PFC (right dorsolateral = RDLPFC, left dorsolateral = LDLPFC, right anterior = RANTPFC, and left anterior = LANTPFC). 

Both the behavioral and fNIRS data were analyzed by using the NCSS statistics software tool. The first step was to screen the data for missing values and test the assumptions of factorial ANOVA such as checking for outliers, normality, and the assumption of sphericity. Normality was tested by conducting Shapiro−Wilk’s test. Potential outliers were checked by conducting descriptive statistical analysis with normality plots. The assumption of sphericity was tested by running Mauchly’s test. To address variability issues, z-scores or standardized scores were computed and plotted for each dependent measure. This was done by using the formula V − X_g_/SD_g_, where V is the variable score (e.g., TT, RT, LDLPFC, etc.), X_g_ is the grand mean for each problem, and SD_g_ is the grand standard deviation for each problem. Z scores were then used in all the subsequent statistical analyses (see Appendix D). 

To test the hypothesis, several repeated-measures factorial ANOVA models were developed and tested separately for each dependent variable. 3 × 2 × 2 repeated-measures factorial ANOVA (Level × Problem × Week) was applied separately on the dependent variables of RT, TT, the OXY biomarker for the four regions of interest (LDLPFC, RDLPCF, LANTPFC, RANTPFC) and the TLX score. The within subject factors of Week, Problem, and Level were fixed effects, while the Subject factor was a random effect. A Greenhouse−Geisser correction was applied when the assumption of sphericity was violated. The alpha level of significance was set at 0.05. Tukey HSD post hoc test was conducted to assess any significant interaction effects. Partial eta-squared (η^2^) was reported for the effect sizes. Cohen’s [37] benchmarks for defining small (η^2^ = 0.01), medium (η^2^ = 0.06) and large (η^2^ = 0.14) effects were used to assist with the interpretation of the calculated effect sizes [21].

## 3. Results

### 3.1. Behavioral Results

**Figure 6:**
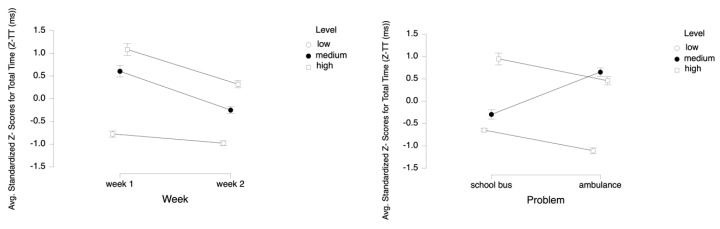
Line plots represent interaction effects of Level and Week (left) and Level and Problem (right) on average standardized z scores for total time (Z-TT) with the error bars representing the standard error of the mean statistic. Differences in average Z-TT (ms) are represented across low, medium, and high cognitive load tasks for Week 1 and Week 2 and for School Bus and Ambulance problem scenarios.

For the dependent variable of TT, the results of the three-way ANOVA revealed two-way interaction effects of Week and Level, *F* (2, 52) = 14.71, *p* ≤ 0.001, η^2^ = 0.36, and Problem and Level, *F* (2, 52) = 93.28, *p* ≤ 0.001, η^2^ = 0.78 (see Figure 6). There were also main effects of Week, *F* (1, 26) = 95.34, *p* ≤ 0.001, η^2^ = 0.79 and Level, *F* (2, 52) = 289.81, *p* ≤ 0.001, η^2^ = 0.92.

**Figure 7:**
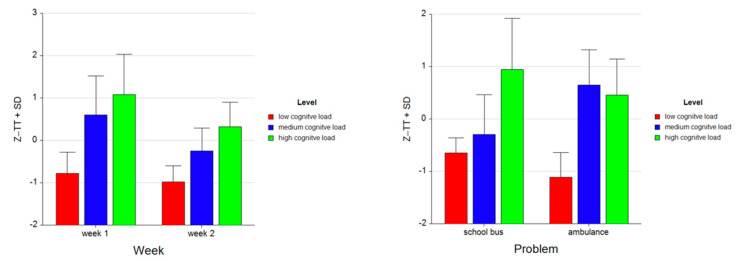
Bar charts represent the average standardized z-scores for total time (Z-TT) and standard deviation (SD) for low cognitive load, medium cognitive load, and high cognitive load tasks for Week 1 and Week 2 (left) and for School Bus and Ambulance problem scenarios (right).

Tukey HSD post hoc tests revealed that the total time spent on the tasks increased as the difficulty level increased both for Week 1 (M (mean) = −0.777, SD (standard deviation) = 0.496 for low, M = 0.604, SD = 0.912 for medium, M = 1.085, SD = 0.948 for high levels) and for Week 2 (M= −0.980, SD = 0.376 for low, M = −0.251; SD = 0.536 for medium, M = 0.320, SD = 0.581 for high levels) (see Figure 7). Overall, the total time spent on both problems decreased from Week 1 (M = 0.304, SD = 1.130) to Week 2 (M = −0.303, SD = 0.733).

**Figure 8:**
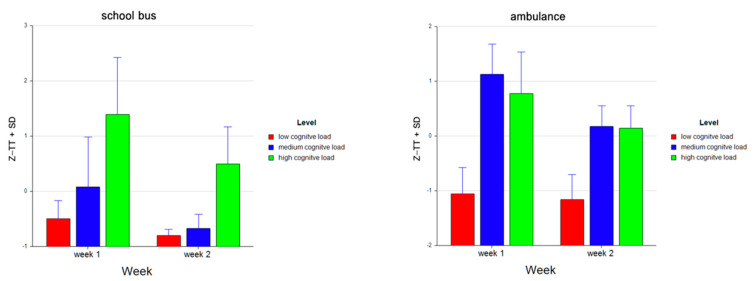
Bar charts represent the average standardized z-scores for total time (Z-TT) and standard deviation (SD) for low cognitive load, medium cognitive load, and high cognitive load tasks for School Bus (left) and Ambulance (right) problem scenarios for Week 1 and Week 2.

For the School Bus problem, it was found that the total time increased as the difficulty level increased (M = −0.649, SD = 0.286 for low, M = −0.297, SD = 0.763 for medium, M = 0.946, SD = 0.971 for high levels). For Ambulance problem, participants spent more time on medium (M = 0.650, SD = 0.673) than on low (M = −1.108, SD = 0.468) and high level tasks (M = 0.458, SD = 0.686). Overall, as the difficulty level increased, the total time spent on the tasks increased as well (M = −0.879, SD = 0.450 for low, M = 0.176, SD = 0.859 for medium, and M= 0.702, SD = 0.872 for high level tasks (see Figure 7). A similar pattern is observed within each week for both the School Bus problem (M = −0.498, SD = 0.327 for low, M = 0.079, SD = 0.907 for medium, M = 1.395, SD = 1.025 for high level tasks in Week 1; M = −0.800, SD = 0.114 for low, M = −0.673, SD = 0.261 for medium, M = 0.498, SD = 0.672 for high level tasks in Week 2) and the Ambulance problem (M = −1.057, SD = 0.481 for low, M = 1.128, SD = 0.550 for medium, M = 0.775, SD = 0.762 for high level tasks in Week 1; M = −1.160, SD = 0.457 for low, M = 0.172, SD = 0.382 for medium, M = 0.141, SD = 0.412 for high level tasks in Week 2) (see Figure 8).

**Figure 9:**
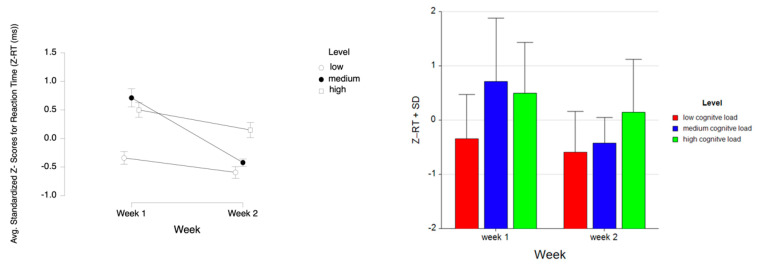
Line plot (left) represents interaction effect of Level and Week on average standardized z scores for reaction time (Z-RT) with the error bars representing the standard error of the mean statistic. Bar chart (right) represents average standardized z-scores for Z-RT and standard deviation (SD) for low cognitive load, medium cognitive load, and high cognitive load tasks for Week 1 and Week 2.

For the dependent measure of RT, the results of the three-way ANOVA revealed a two-way interaction effect of Week and Level, *F* (2, 52) = 12.19, *p* ≤ 0.001, η^2^ = 0.32. There were also main effects of Week, *F* (1, 26) = 46.46, *p* ≤ 0.001, η^2^ = 0.64 and Level, *F* (2, 52) = 30.42, *p* ≤ 0.001, η^2^ = 0.54. Tukey HSD post hoc tests revealed that the reaction time increased as the difficulty level increased both for Week 1 (M = −0.777, SD = 0.496 for low, M = 0.604, SD = 0.912 for medium, M = 1.085, SD = 0.948 for high level tasks) and Week 2 (M = −0.980, SD = 0.376 for low, M = −0.251, SD = 0.536 for medium, M = 0.320, SD = 0.581 for high level tasks) (see Figure 9). It was also found that there was an overall decrease in reaction time from Week 1 (M = 0.304, SD = 1.130) to Week 2 (M = −0.304, SD = 0.733). 

### 3.2. fNIRS Results

**Figure 10:**
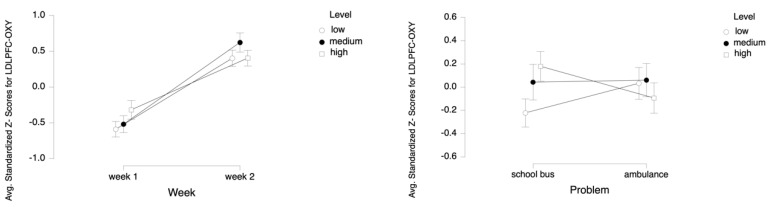
Line plots represent interaction effects of Level and Week (left) and Level and Problem (right) on average standardized z scores for oxygenation for left dorsolateral prefrontal cortex (Z-LDLPFC_OXY) with the error bars representing the standard error of the mean statistic. Differences in average oxygenation Z-LDLPFC_OXY are represented across low, medium, and high cognitive load tasks for Week 1 and Week 2 and for School Bus and Ambulance problem scenarios.

For the dependent measure of LDLPFC OXY, the results of the three-way ANOVA revealed two-way interaction effects of Week and Level, *F* (2, 52) = 6.92, *p* ≤ 0.05, η^2^ = 0.21, as well as Problem and Level, *F* (2, 52) = 13.23, *p* ≤ 0.001, η^2^ = 0.34 (see Figure 10). There were also main effects of Week, *F* (1, 26) = 32.58, *p* ≤ 0.001, η^2^ = 0.56 and Level, *F* (2, 52) = 4.23, *p* ≤ 0.05, η^2^ = 0.14.

**Figure 11:**
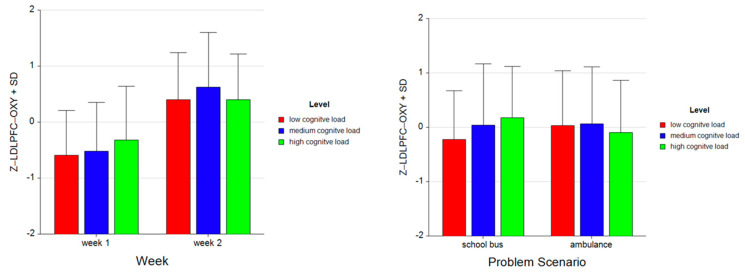
Bar charts represent average standardized z-scores for oxygenation for left dorsolateral prefrontal cortex (Z-LDLPFC-OXY) and standard deviation (SD) for low cognitive load, medium cognitive load, and high cognitive load tasks for experiment Week 1 and Week 2 (left) and School Bus and Ambulance problem scenarios (right).

**Figure 12:**
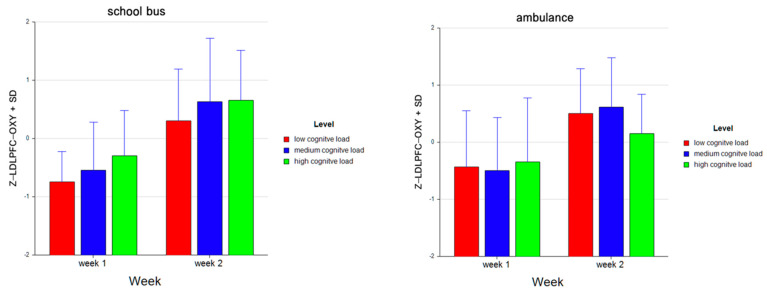
Bar charts represent the average standardized z-scores for oxygenation for left dorsolateral prefrontal cortex (Z-LDLPFC-OXY) and standard deviation (SD) for low cognitive load, medium cognitive load, and high cognitive load tasks for School Bus (left) and Ambulance (right) problem scenarios for Week 1 and Week 2.

Tukey HSD post hoc tests revealed that the average oxygenation for the LDLPFC region increased as the difficulty level of the tasks increased for Week 1 (M = −0.590, SD = 0.798 for low, M = −0.519, SD = 0.871 for medium, M = −0.318, SD = 0.955 for high level tasks). For Week 2, average oxygenation for medium level tasks was higher (M = 0.623, SD = 0.974) as compared to low (M = 0.401, SD = 0.838) and high (M = 0.404, SD = 0.811) level tasks. Overall, there was an increase in average oxygenation from Week 1 (M = −0.476, SD = 0.879) to Week 2 (M = 0.476, SD = 0.878). For the School Bus problem, it was found that average oxygenation for the LDLPFC region increased as the difficulty level increased (M = −0.222, SD = 0.894 for low, M = 0.043, SD = 1.126 for medium, M = 0.179, SD = 0.941 for high level tasks). For the Ambulance Problem, it was found that the average oxygenation level was highest for medium level tasks (M = 0.060, SD = 1.084), followed by low (M = 0.033, SD = 1.004) and high (M = −0.093, SD = 0.955) level tasks. Overall, oxygenation was highest for the medium level tasks (M = 0.052, SD = 1.084), followed by high (M = 0.043, SD = 0.954) and low level tasks (M = −0.094, SD = 0.955) (see Figure 11). Similarly, an increasing pattern is observed from Week 1 to Week 2 within each level for each problem separately: the School Bus problem (M = −0.745, SD = 0.518 for low, M = −0.542, SD = 0.825 for medium, M = −0.294, SD = 0.776 for high level tasks for Week 1, M = 0.301, SD = 0.890 for low, M = 0.628, SD = 1.092 for medium, M = 0.653, SD = 0.860 for high level tasks for Week 2) and the Ambulance problem (M = −0.435, SD = 0.990 for low, M = −0.496, SD = 0.931 for medium, M = −0.342, SD = 1.121 for high level tasks for Week 1, M = 0.501, SD = 0.787 for low, M = 0.617, SD = 0.861 for medium, M = 0.155, SD = 0.688 for high level tasks for Week 2) (see Figure 12). 

**Figure 13:**
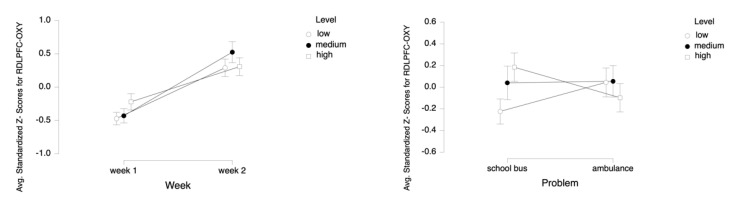
Line plots represent interaction effects of Level and Week (left) and Level and Problem (right) on average standardized z scores for oxygenation for right dorsolateral prefrontal cortex (Z-RDLPFC-OXY) with the error bars representing the standard error of the mean statistic. Differences in average oxygenation for Z-RDLPFC are represented across low, medium, and high cognitive load tasks for Week 1 and Week 2 and for School Bus and Ambulance problem scenarios.

For the dependent measure of RDLPFC OXY, the results of the three-way ANOVA revealed two-way interaction effects of Week and Level, *F* (2, 52) = 7.18, *p* ≤ 0.05, η^2^ = 0.22, as well as Problem and Level, *F* (2, 52) = 18.07, *p* ≤ 0.00, η^2^ = 0.41 (see Figure 13). There were also main effects of Week, *F* (1, 26) = 16.31, *p* ≤ 0.001, η^2^ = 0.39 and Level, *F* (2, 52) = 4.05, *p* ≤ 0.05, η^2^ = 0.14.

**Figure 14:**
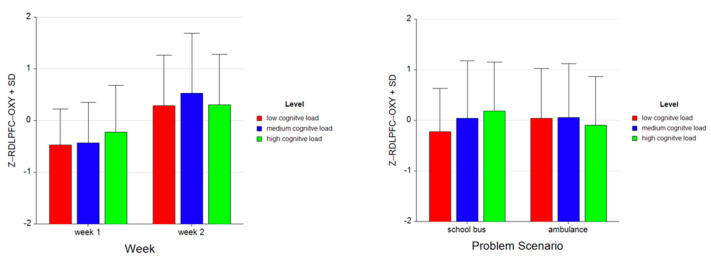
Bar charts represent average standardized z-scores for oxygenation for right dorsolateral prefrontal cortex (Z-RDLPFC-OXY) and standard deviation (SD) for low cognitive load, medium cognitive load, and high cognitive load tasks for experiment Week 1 and Week 2 (left) and School Bus and Ambulance problem scenarios (right).

**Figure 15:**
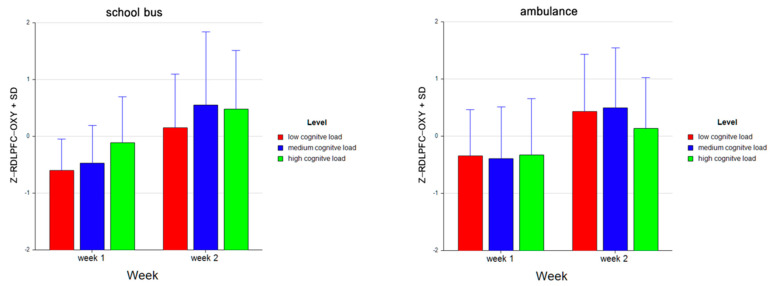
Bar charts represent the average standardized z-scores for oxygenation for right dorsolateral prefrontal cortex (Z-RDLPFC-OXY) and standard deviation (SD) for low cognitive load, medium cognitive load, and high cognitive load tasks for School Bus (left) and Ambulance (right) problem scenarios for Week 1 and Week 2.

Tukey HSD post hoc tests revealed that, for Week 1, average oxygenation for the RDLPFC region increased as the difficulty level of the tasks increased for Week 1 (M = −0.471, SD = 0.696 for low, M = −0.430, SD = 0.784 for medium, M = −0.220, SD = 0.901 for high level tasks). For Week 2, average oxygenation was higher for medium level tasks (M = 0.524, SD = 1.163), followed by high (M = 0.307, SD = 0.970) and low (M = 0.290, SD = 0.971) level tasks. Overall, there was an increase in average oxygenation from Week 1 (M = −0.374, SD = 0.801) to Week 2 (M = 0.374, SD = 1.038). For the School Bus problem, it was found that average oxygenation for the RDLPFC region increased as the difficulty level increased (M = −0.225, SD = 0.853 for low, M = 0.040, SD = 1.137 for medium, M = 0.184, SD = 0.964 for high level tasks). For the Ambulance Problem, it was found that the average oxygenation level was highest for medium tasks (M = 0.054, SD = 1.067), followed by low (M = 0.044, SD = 0.980) and high level tasks (M = −0.098, SD = 0.961). Overall, oxygenation was highest for the medium level tasks (M = 0.047, SD = 1.098), followed by high (M = 0.043, SD = 0.969) and low level tasks (M = −0.090, SD = 0.924) (see Figure 14). Similarly, an increasing pattern is observed from Week 1 to Week 2 within each level for each problem separately: the School Bus problem (M = −0.602, SD = 0.551 for low, M = −0.471, SD = 0.660 for medium, M = −0.110, SD = 0.807 for high level tasks for Week 1, M = 0.153, SD = 0.940 for low, M = 0.556, SD = 1.287 for medium, M = 0.479, SD = 1.031 for high level tasks for Week 2) and the Ambulance problem (M = −0.341, SD = 0.806 for low, M = −0.388, SD = 0.902 for medium, M = −0.330, SD = 0.988 for high level tasks for Week 1, M = 0.428, SD = 1.000 for low, M = 0.497, SD = 1.050 for medium, M = 0.135, SD = 0.891 for high level tasks for Week 2) (see Figure 15).

**Figure 16:**
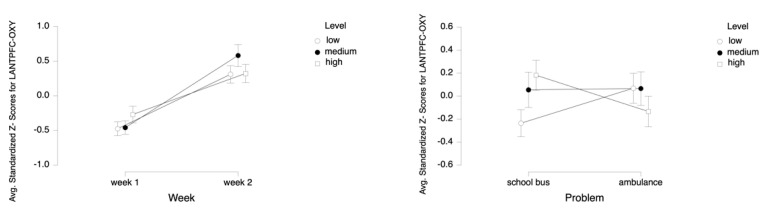
Line plots represent interaction effects of Level and Week (left) and Level and Problem (right) on average standardized z scores for oxygenation for left anterior prefrontal cortex (Z-LANTPFC-OXY) with the error bars representing the standard error of the mean statistic. Differences in average oxygenation for Z-LANTPFC are represented across low, medium, and high cognitive load tasks for Week 1 and Week 2 and for School Bus and Ambulance problem scenarios.

For the dependent measure of LANTPFC OXY, the results of the three-way ANOVA revealed two-way interaction effects of Week and Level, *F* (2, 52) = 5.27, *p* ≤ 0.05, η^2^ = 0.17 (Figure 16), as well as Problem and Level, *F* (2, 52) = 15.52, *p* ≤ 0.001, η^2^ = 0.37 (see Figure 13). There were also main effects of Week, *F* (1, 26) = 29.62, *p* ≤ 0.001, η^2^ = 0.53 and Level, *F* (2, 52) = 3.75, *p* ≤ 0.05, η^2^ = 0.13.

**Figure 17:**
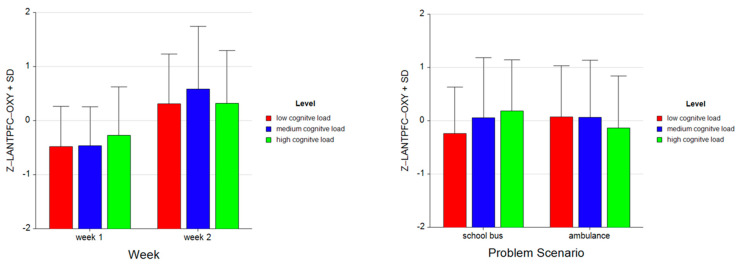
Bar charts represent average standardized z-scores for oxygenation for left anterior prefrontal cortex (Z-LANTPFC-OXY) and standard deviation (SD) for low cognitive load, medium cognitive load, and high cognitive load tasks for experiment Week 1 and Week 2 (left) and School Bus and Ambulance problem scenarios (right).

**Figure 18:**
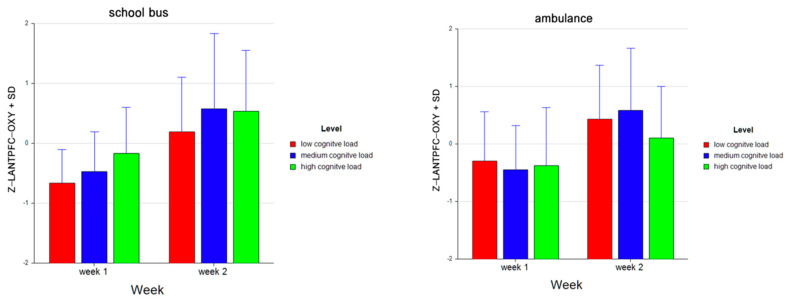
Bar charts represent the average standardized z-scores for oxygenation for left anterior prefrontal cortex (Z-LANTPFC-OXY) and standard deviation (SD) for low cognitive load, medium cognitive load, and high cognitive load tasks for School Bus (left) and Ambulance (right) problem scenarios for Week 1 and Week 2.

Tukey HSD post hoc tests revealed that, for Week 1, average oxygenation for the LANTPFC region increased as the difficulty level of the tasks increased for Week 1 (M = −0.477, SD = 0.737 for low, M = −0.460, SD = 0.715 for medium, M = −0.272, SD = 0.896 for high level tasks). For Week 2, the average oxygenation was higher for medium level tasks (M = 0.581, SD = 1.161), followed by high (M = 0.320, SD = 0.972) and low (M = 0.309, SD = 0.927) level tasks. Overall, there was an increase in average oxygenation from Week 1 (M = −0.403, SD = 0.787) to Week 2 (M = 0.403, SD = 1.026). For the School Bus problem, it was found that average oxygenation for the LANTPFC region increased as the difficulty level increased (M = −0.236, SD = 0.864 for low, M = 0.054, SD = 1.126 for medium, M = 0.182, SD = 0.964 for high level tasks). For the Ambulance Problem, it was found that the average oxygenation level was highest for low level tasks (M = 0.068, SD = 0.960), followed by medium (M = 0.066, SD = 1.069) and high level tasks (M = −0.134, SD = 0.973). Overall, oxygenation was highest for the medium level tasks (M = 0.060, SD = 1.093), followed by high (M = 0.024, SD = 0.977) and low (M = −0.084, SD = 0.922) level tasks (see Figure 17). Similarly, an increasing pattern is observed from Week 1 to Week 2 within each level for each problem separately: the School Bus problem (M = −0.661, SD = 0.554 for low, M = −0.469 SD = 0.664 for medium, M = −0.169, SD = 0.773 for high level tasks for Week 1, M = 0.189, SD = 0.917 for low, M = 0.578, SD = 1.255 for medium, M = 0.533, SD = 1.020 for high level tasks for Week 2) and the Ambulance problem (M = −0.292, SD = 0.854 for low, M = −0.452, SD = 0.775 for medium, M = −0.375, SD = 1.008 for high level tasks for Week 1, M = 0.429, SD = 0.938 for low, M = 0.583, SD = 1.082 for medium, M = 0.108, SD = 0.890 for high level tasks for Week 2) (see Figure 18).

**Figure 19:**
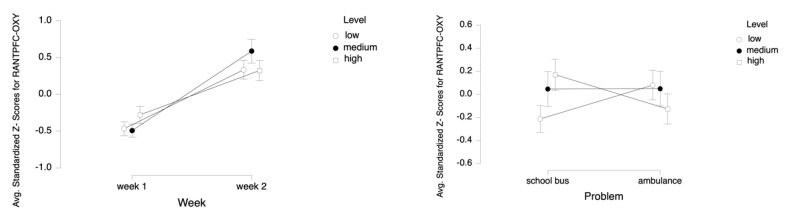
Line plots represent interaction effects of Level and Week (left) and Level and Problem (right) on average standardized z scores for oxygenation for right anterior prefrontal cortex (Z-RANTPFC-OXY) with the error bars representing the standard error of the mean statistic. Differences in average oxygenation for Z-RANTPFC are represented across low, medium, and high cognitive load tasks for Week 1 and Week 2 and for School Bus and Ambulance problem scenarios.

For the dependent measure of RANTPFC OXY, the results of the three-way ANOVA revealed two-way interaction effects of Week and Level, *F* (2, 52) = 7.47, *p* ≤ 0.00, η^2^ = 0.22, as well as Problem and Level, *F* (2, 52) = 15.42, *p* ≤ 0.001, η^2^ = 0.37 (see Figure 19). There was also a main effect of Week, *F* (1, 26) = 25.92, *p* ≤ 0.001, η^2^ = 0.50.

**Figure 20:**
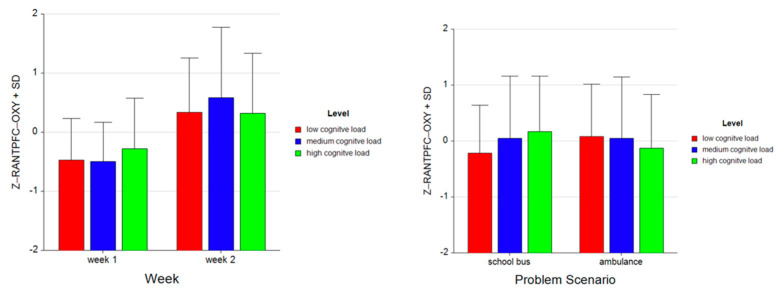
Bar charts represent average standardized z-scores for oxygenation for right anterior prefrontal cortex (Z-RANTPFC-OXY) and standard deviation (SD) for low cognitive load, medium cognitive load, and high cognitive load tasks for experiment Week 1 and Week 2 (left) and School Bus and Ambulance problem scenarios (right).

**Figure 21:**
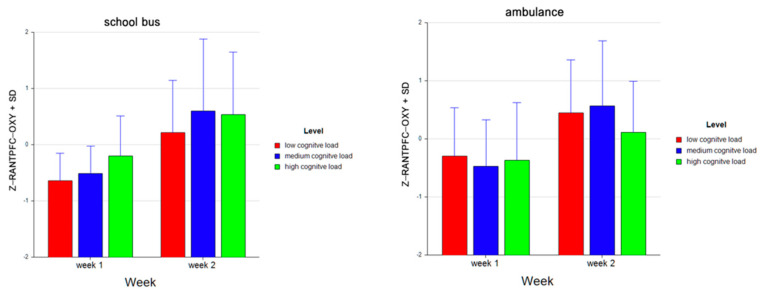
Bar charts represent the average standardized z-scores for oxygenation for right anterior prefrontal cortex (Z-RANTPFC-OXY) and standard deviation (SD) for low cognitive load, medium cognitive load, and high cognitive load tasks for School Bus (left) and Ambulance (right) problem scenarios for Week 1 and Week 2.

Tukey HSD post hoc tests revealed that, for Week 1, average oxygenation for the RANTPFC region increased as the difficulty level of the tasks increased for Week 1 (M =−0.468, SD =0.697 for low, M = −0.493, SD = 0.660 for medium, M = −0.281, SD = 0.857 for high level tasks). For Week 2, average oxygenation was higher for medium level tasks (M = 0.587, SD = 1.161), followed by low (M = 0.333, SD = 0.922) and high (M = 0.322, SD = 1.016) level tasks. Overall, there was an increase in average oxygenation from Week 1 (M = −0.414, SD = 0.745) to Week 2 (M = 0.414, SD = 1.049). For the School Bus problem, it was found that average oxygenation for the RANTPFC region increased as the difficulty level increased (M = −0.214, SD = 0.857 for low, M = 0.046, SD = 1.112 for medium, M = 0.169, SD = 0.995 for high level tasks). For the Ambulance problem, it was found that the average oxygenation level was highest for low level tasks (M = 0.079, SD = 0.941), followed by medium (M = 0.048, SD = 1.099) and high level tasks (M = −0.127, SD = 0.958) (see Figure 20). Similarly, an increasing pattern is observed from Week 1 to Week 2 within each level for each problem separately: the School Bus problem (M = −0.644, SD = 0.493 for low, M = −0.511, SD = 0.491 for medium, M = −0.197, SD = 0.708 for high level tasks for Week 1, M = 0.215, SD = 0.933 for low, M = 0.603, SD = 1.279 for medium, M = 0.534, SD = 1.112 for high level tasks for Week 2) and the Ambulance problem (M = −0.292, SD = 0.827 for low, M = −0.474, SD = 0.803 for medium, M = −0.365, SD = 0.991 for high level tasks for Week 1, M = 0.450, SD = 0.914 for low, M = 0.571, SD =1.118 for medium, M = 0.110, SD = 0.879 for high level tasks for Week 2) (see Figure 21).

### 3.3. Perceived Mental Workload Results

**Figure 22:**
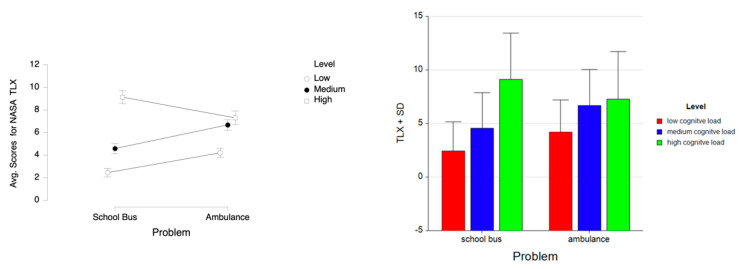
Line plot (left) represents interaction effect of Problem and Level on average scores for perceived mental workload as measured by NASA TLX with the error bars representing the standard error of the mean statistic. Bar chart (right) represents average scores for NASA TLX and standard deviation (SD) for low cognitive load, medium cognitive load, and high cognitive load tasks for School Bus (left) and Ambulance (right) problem scenarios.

**Figure 23:**
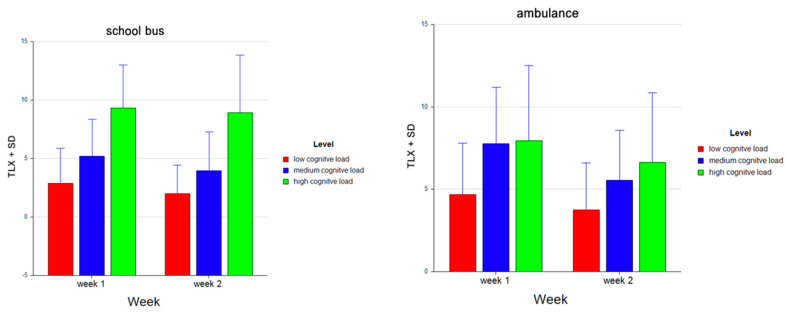
Bar charts represent the average standardized z-scores for oxygenation for perceived mental workload as measured by NASA TLX and standard deviation (SD) for low cognitive load, medium cognitive load, and high cognitive load tasks for School Bus (left) and Ambulance (right) problem scenarios for Week 1 and Week 2.

For the dependent measure of perceived mental workload, as measured by NASA TLX instrument, the results of the three-way ANOVA revealed a two-way interaction effect of Problem and Level, *F* (2, 52) = 20.90, *p* ≤ 0.001, η^2^ = 0.45. There were main effects of Level, *F* (2, 52) = 66.72, *p* ≤ 0.001, η^2^ = 0.72 and Week, *F* (1, 26) = 9.39, *p* ≤ 0.05, η^2^ = 0.27. Tukey HSD post hoc tests revealed that the NASA TLX score increased as the difficulty level increased both for the School Bus problem (M = 2.444, SD = 2.731 for low, M = 4.574, SD = 3.289 for medium, M = 9.130, SD = 4.296 for high level) and the Ambulance problem (M = 4.204, SD = 2.999 for low, M = 6.667; SD = 3.381 for medium, M = 7.296, SD = 4.407 for high level tasks). Overall, the NASA TLX scores for the School Bus problem were lower (M = 5.383, SD = 4.463) than those for the Ambulance problem (M = 6.056, SD = 3.861). It was also found that, overall, the perceived mental workload decreased from Week 1 (M = 6.302, SD = 4.129) to Week 2 (M = 5.136, SD = 4.162) for both problems combined (see Figure 22). A similar pattern is observed within each week for both the School Bus problem (M = 2.889, SD = 2.979 for low, M = 5.185, SD = 3.187 for medium, M = 9.333, SD = 3.679 for high level tasks in Week 1; M = 2.000, SD = 2.434 for low, M = 3.963, SD = 3.334 for medium, M = 8.926, SD = 4.898 for high level tasks in Week 2) and the Ambulance problem (M = 4.667, SD = 3.126 for low, M = 7.778, SD = 3.412 for medium, M = 7.963, SD = 4.553 for high level tasks in Week 1; M = 3.741, SD = 2.850 for low, M = 5.556, SD = 3.017 for medium, M = 6.627, SD = 4.235 for high level tasks in Week 2) (see Figure 23).

### 3.4. Results Summary

The results of the statistical analyses revealed a number of interaction effects and main effects for the behavioral, fNIRS, and NASA TLX data. Table 1 below summarizes these interaction effects and main effects for each dependent measure.

## 4. Discussion

This study used a hybrid approach to assessing mental workload [4,22] where we combined (1) task performance measures such as total time spent on a task (TT) and reaction time (RT); (2) neurophysiological measures involving the use of functional near-infrared spectroscopy (fNIRS) and a subjective rating scale for self-assessment of mental workload (NASA TLX).The results confirmed that there are interaction effects between Week, Problem, and Level on performance as measured by reaction time and total time. Furthermore, the associated effect sizes for the behavioral measures ranged from η^2^ = 0.32∓0.92 indicating a large effect [37]. The results showed that, overall, an improved performance was observed for the two analogous problem-solving tasks in Week 2 as compared to Week 1 of the experiment across all the three difficulty levels of the tasks. This finding suggests that the participants were able to retain and transfer their problem-solving skills from Week 1 to Week 2. Moreover, analyses indicated that participant performance was sensitive to task difficulty, showing a longer reaction time and total time for medium and high cognitive load tasks as compared to low cognitive load tasks. These findings are consistent with previous studies showing that task features, such as working memory demands and semantic content, affect behavioral performance and transfer during analogical problem solving [6,38,39]. 

The results confirmed the main hypothesis stating that there are interaction effects between Week, Problem, and Level on mental workload as measured by OXY (µV). Several significant interaction effects for each of the four regions of interest were revealed. Furthermore, the associated effect sizes for the OXY biomarker ranged from η^2^ = 0.13 − 0.26 indicating medium to large effects [37]. The results corroborate the findings from previous fNIRS studies on mental workload changes during cognitive tasks, confirming that mental workload was sensitive to task difficulty, with small to moderate effect sizes for the OXY biomarker [1,25]. The results from this study showed that all the four brain regions of interest were sensitive to workload changes. These findings are in parallel with previous studies with functional brain imaging techniques confirming that different brain regions within the PFC area are sensitive to task manipulations during various cognitive tasks [1,6,7,8,27,33]. In particular, our study results indicated that for both the Ambulance and School Bus scenarios, bilateral activation was observed both in the left (LDLPFC, LANTPFC) and in the right (RDLPFC, RANTPFC). This finding is not surprising given that the SNT game tasks involved cognitive processes of problem solving, analogical reasoning, spatial navigation, planning, and decision making, which, as discussed earlier, are associated with both hemispheres of PFC. This finding is consistent with the fNIRS study conducted by Liang and colleagues [1] which used the Tower of Hanoi tasks to measure mental workload changes across the four brain regions of interests in PFC. The findings from the Liang and colleagues [1] study showed a bilateral activation across the left and the right PFC regions reflected by the biomarkers of OXY and oxy-Hb. 

The findings from the behavioral and neural data analyses contribute to the existing body of research within CLT which informs educational practice by proposing solutions based on human cognitive architecture [10]. In particular, this investigation with a controlled serious gaming environment provided empirical evidence, both at behavioral and neural levels, that the subjects were sensitive to the intrinsic cognitive load imposed by the task difficulty, as well as extraneous load imposed by two different problem scenarios (i.e., Ambulance and School Bus). In particular, for the School Bus problem, an overall monotonic increase across behavioral and neural measures was observed as the difficulty level of the tasks increased. However, in the case of the Ambulance problem, the changes across the behavioral and neural measures in response to the increased task difficulty were inconsistent compared to the School Bus problem. This inconsistency could be explained by the differences in the contextual features of the problem scenarios (e.g., driving a school bus at daytime to pick up students (School Bus problem) vs. driving an ambulance at night to pick up patients (Ambulance Problem)). These contextual differences, although not directly related to the intrinsic nature of the tasks (i.e., intrinsic cognitive load), could potentially exert different extraneous loads on the subject. Further research with the deployment of fNIRS could provide a more in-depth understanding of how various contextual manipulations of analogous complex cognitive tasks affect mental workload and behavioral performance across varying difficulty levels.

One inconsistency between the neural and behavioral data relates to the overall increase in average oxygenation for each of the four regions of interest compared to the overall decrease in performance measures from Week 1 to Week 2 for both problem scenarios. One possible explanation for this inconsistency could be that the positive transfer at the behavioral level manifested by a reduced total time and reaction time from Week 1 to Week 2 was at the cost of increased mental workload. This finding contradicts previous studies with fNIRS reporting improved performance (e.g., a decrease in total time required to complete a task) accompanied by reduced activity in the PFC during the later stages of learning [1,4,28]. It is possible that making the tasks within each level slightly more challenging in Week 2 (e.g., having one additional collectable or slightly less fuel) required more cognitive effort on part of the subjects, although at the behavioral level, the subjects performed better in Week 2 compared to Week 1 for both the School Bus and Ambulance problems (as indicated by a decrease in TT and RT). Furthermore, the NASA TLX results for both the School Bus and Ambulance problems were consistent with the behavioral measures, indicating that within each level the subjects perceived the tasks in Week 2 to be less mentally demanding than in Week 1. The findings suggest that there might be a compensatory mechanism at play allowing for improved performance at the expense of increased mental workload. This explanation is supported by the compensatory control model proposed by Hockey [40] stating that effective performance under high demands is accompanied by high levels of physiological activation. According to this model, performance protection requires compensatory costs such as increased mental effort. This is viewed as a trade-off between maintenance of the primary task goals and the amount of mental effort to be invested in the task. Future empirical research is needed to confirm this explanation and provide a deeper understanding on various compensatory mechanisms involved in the maintenance of performance under varying task demands.

## 5. Conclusions

This study investigated mental workload during the SNT game involving complex cognitive tasks of varying workload demands. A number of significant interaction effects were found across the behavioral measures of TT, RT, the neural measures of OXY (µV) for each of the four regions of interest within PFC (LDLPFC, RDLPFC, LANTPFC, RANTPFC) as well as the NASA TLX measure of perceived mental workload. The results confirmed the main hypothesis stating that there are interaction effects between Week, Problem, and Level on mental workload as measured by OXY (µV). The current study presents a promising application of fNIRS for measuring hemodynamic changes within PFC under different workload conditions in the context of serious gaming.

## Figures and Tables

**Table 1:** Summary of repeated-measures factorial ANOVA results with interaction effects and main effects.

DV	Effect	F(df)	*p*-Value	η^2^
TT	Week × Level	*F* (2, 52) = 14.71	<0.001	0.36
	Problem × Level	*F* (2, 52) = 93.28,	<0.001	0.78
	Week	*F* (1, 26) = 95.34	<0.001	0.79
	Level	*F* (2, 52) = 289.81	<0.001	0.92
RT	Week × Level	*F* (2, 52) = 12.19	<0.001	0.32
	Week	*F* (1, 26) = 46.46	<0.001	0.64
	Level	*F* (2, 52) = 30.42	<0.001	0.54
LDLPFC	Week × Level	*F* (2, 52) = 6.92	<0.05	0.21
	Problem × Level	*F* (2, 52) = 13.23	<0.001	0.34
	Week	*F* (1, 26) = 32.58	<0.001	0.56
	Level	*F* (2, 52) = 4.23	<0.05	0.14
RDLPFC	Week × Level	*F* (2, 52) = 7.18	<0.05	0.22
	Problem × Level	*F* (2, 52) = 18.07	<0.001	0.41
	Week	*F* (1, 26) = 16.31	<0.001	0.39
	Level	*F* (2, 52) = 4.05	<0.05	0.14
LANTPFC	Week × Level	*F* (2, 52) = 5.27	<0.05	0.17
	Problem × Level	*F* (2, 52) = 15.52	<0.001	0.37
	Week	*F* (1, 26) = 29.62	<0.001	0.53
	Level	*F* (2, 52) = 3.75	<0.05	0.13
RANTPFC	Week × Level	*F* (2, 52) = 7.47	<0.05	0.22
	Problem × Level	*F* (2, 52) = 15.42	<0.001	0.37
	Week	*F* (1, 26) = 25.92	<0.001	0.50
TLX	Problem × Level	*F* (2, 52) = 20.90	<0.001	0.45
	Level	*F* (2, 52) = 66.72	<0.001	0.72
	Week	*F* (1, 26) = 9.39	<0.05	0.27

**Note.** Dependent Variables (DV) represent average standardized z scores for total time (TT), reaction time (RT) and oxygenation (OXY) for left dorsolateral prefrontal cortex (LDLPFC), right dorsolateral prefrontal cortex (RDLPFC), left anterior prefrontal cortex (LANTPFC), and right anterior prefrontal cortex (RANTPFC), and the scores for perceived mental workload as measured by NASA TLX.

**Figure A1:**
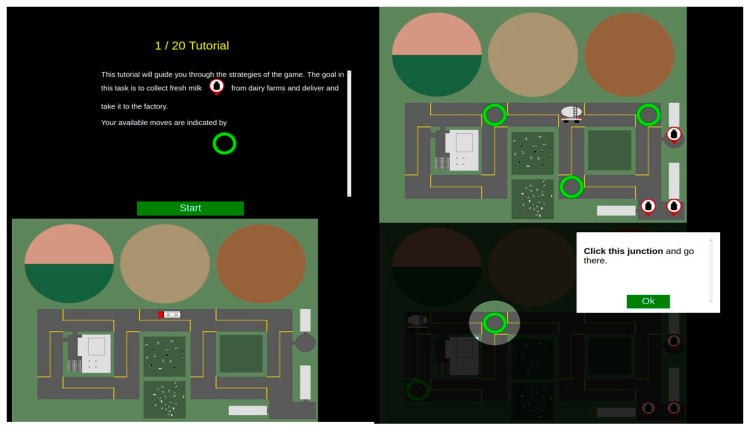
Screenshots of the tutorial with the instruction page.

## Data Availability

The data presented in this study are available on request from the corresponding author. The data are not publicly available due to privacy restrictions.

## Appendix A

The selection criteria for participating in the experiment are listed below:

- Enrolled in the full-time or part-time graduate degree program
- 18 years old or older
- Right-handed, with vision correctible to 20/20
- Do not have history of seizures, head injury, or neurological dysfunction (e.g., Stroke or seizure)
- Do not have a history of depression, schizophrenia, or social phobia.
- Do not have previous admission to an alcohol/drug treatment program or diagnosis of alcohol/drug abuse.
- Are not take any medication known to affect alertness/brain activity (e.g., sleeping pills, Valium, or Xanax).
- Is not pregnant.

## Appendix B

**Table A1:** Descriptive statistics with normality tests for reaction time (RT) (ms) and Total Time (TT) (ms) for the tutorial.

	RT (ms)	TT (ms)
Valid	27	27
Missing	0	0
Mean	4770.222	24,358.889
Median	4400.000	23,599.000
Std. Deviation	1778.093	7670.567
Skewness	0.952	1.130
Std. Error of Skewness	0.448	0.448
Kurtosis	1.060	1.239
Std. Error of Kurtosis	0.872	0.872
Shapiro-Wilk	0.937	0.898
*p*-value of Shapiro-Wilk	0.106	0.012
Range	7608.000	29,795.000

## Appendix D

**Table A2:** Week 1 standardized Z scores for School Bus and Ambulance problem scenarios.

DV	Week 1 Mean ± SD					
	School Bus			Ambulance		
	Low	Mid	High	Low	Mid	High
Z_RT	−0.279 ± 0.345	0.668 ± 1.536	0.531 ± 0.866	−0.402 ± 1.105	0.754 ± 0.636	0.468 ± 1.006
Z_TT	−0.498 ± 0.327	0.079 ± 0.907	1.395 ± 1.025	−1.057 ± 0.481	1.128 ± 0.550	0.775 ± 0.762
Z_LDLPFC	−0.745 ± 0.518	−0.542 ± 0.824	−0.294 ± 0.775	−0.435 ± 0.989	−0.496 ± 0.930	−0.342 ± 1.121
Z_RDLPFC	−0.602 ± 0.551	−0.471 ± 0.660	−0.110 ± 0.807	−0.340 ± 0.806	−0.388 ± 0.902	−0.330 ± 0.988
Z_LANTPFC	−0.661 ± 0.554	−0.469 ± 0.664	−0.169 ± 0.773	−0.292 ± 0.854	−0.451 ± 0.775	−0.375 ± 1.007
Z_RANTPFC	−0.644 ± 0.493	−0.511 ± 0.491	−0.197 ± 0.708	−0.292 ± 0.827	−0.474 ± 0.803	−0.364 ± 0.991

Note. Means and standard deviations (SD) of standardized z scores were computed for the variables of reaction time (Z_RT), total time (Z_TT), average oxygenation for left dorsolateral prefrontal cortex (Z_LDLPFC), right dorsolateral prefrontal cortex (Z_RDLPFC), left anterior prefrontal cortex (Z_LANTPFC), and right anterior prefrontal cortex (Z_RDLPFC) across three difficulty levels (high cognitive load, medium cognitive load, and high cognitive load) for Week 1.

**Table A3:** Week 2 standardized Z scores for School Bus and Ambulance problem scenarios.

DV	Week 2 Mean ± SD					
	School Bus			Ambulance		
	Low	Mid	High	Low	Mid	High
Z_RT	−0.642 ± 0.129	−0.3948 ± 0.409	0.117 ± 1.156	−0.549 ± 1.067	−0.449 ± 0.527	0.178 ± 0.772
Z_TT	−0.800 ± 0.113	−0.673 ± 0.261	0.498 ± 0.672	−1.159 ± 0.457	0.172 ± 0.382	0.141 ± 0.412
Z_LDLPFC	−0.222 ± 0.894	0.043 ± 1.126	0.179 ± 0.941	0.033 ± 1.003	0.060 ± 1.051	−0.093 ± 0.955
Z_RDLPFC	−0.224 ± 0.853	0.040 ± 1.137	0.184 ± 0.964	0.044 ± 0.979	0.054 ± 1.067	−0.098 ± 0.961
Z_LANTPFC	−0.236 ± 0.864	0.054 ± 1.126	0.181 ± 0.964	0.068 ± 0.960	0.066 ± 1.069	−0.134 ± 0.972
Z_RANTPFC	−0.214 ± 0.856	0.046 ± 1.112	0.169 ± 0.994	0.079 ± 0.941	0.048 ± 1.099	−0.127 ± 0.958

Note. Means and standard deviations (SD) of standardized z scores were computed for the variables of reaction time (Z_RT), total time (Z_TT), average oxygenation for left dorsolateral prefrontal cortex (Z_LDLPFC), right dorsolateral prefrontal cortex (Z_RDLPFC), left anterior prefrontal cortex (Z_LANTPFC), and right anterior prefrontal cortex (Z_RDLPFC) across three difficulty levels (high cognitive load, medium cognitive load, and high cognitive load) for two problem scenarios (School Bus and Ambulance) for Week 2.
